# Comprehensive analysis of a tryptophan metabolism-related model in the prognostic prediction and immune status for clear cell renal carcinoma

**DOI:** 10.1186/s40001-023-01619-0

**Published:** 2024-01-05

**Authors:** Qinfan Yao, Xiuyuan Zhang, Yucheng Wang, Cuili Wang, Chunchun Wei, Jianghua Chen, Dajin Chen

**Affiliations:** 1 Kidney Disease Center, The First Affiliated Hospital, College of Medicine, Zhejiang University, Hangzhou, China; 2 Key Laboratory of Kidney Disease Prevention and Control Technology, Zhejiang Province, Hangzhou, China; 3 Institute of Nephropathy, Zhejiang University, Hangzhou, China; 4 Zhejiang Clinical Research Center of Kidney and Urinary System Disease, Hangzhou, China

**Keywords:** ccRCC, Tryptophan metabolism, Prognosis, Immune features, Risk Score

## Abstract

**Background:**

Clear cell renal cell carcinoma (ccRCC) is characterized as one of the most common types of urological cancer with high degrees of malignancy and mortality. Due to the limited effectiveness of existing traditional therapeutic methods and poor prognosis, the treatment and therapy of advanced ccRCC patients remain challenging. Tryptophan metabolism has been widely investigated because it significantly participates in the malignant traits of multiple cancers. The functions and prognostic values of tryptophan metabolism-related genes (TMR) in ccRCC remain virtually obscure.

**Methods:**

We employed the expression levels of 40 TMR genes to identify the subtypes of ccRCC and explored the clinical characteristics, prognosis, immune features, and immunotherapy response in the subtypes. Then, a model was constructed for the prediction of prognosis based on the differentially expressed genes (DEGs) in the subtypes from the TCGA database and verified using the ICGC database. The prediction performance of this model was confirmed by the receiver operating characteristic (ROC) curves. The relationship of Risk Score with the infiltration of distinct tumor microenvironment cells, the expression profiles of immune checkpoint genes, and the treatment benefits of immunotherapy and chemotherapy drugs were also investigated.

**Results:**

The two subtypes revealed dramatic differences in terms of clinical characteristics, prognosis, immune features, and immunotherapy response. The constructed 6-gene-based model showed that the high Risk Score was significantly connected to poor overall survival (OS) and advanced tumor stages. Furthermore, increased expression of CYP1B1, KMO, and TDO2 was observed in ccRCC tissues at the translation levels, and an unfavorable prognosis for these patients was also found.

**Conclusion:**

We identified 2 molecular subtypes of ccRCC based on the expression of TMR genes and constructed a prognosis-related model that may be used as a powerful tool to guide the prediction of ccRCC prognosis and personalized therapy. In addition, CYP1B1, KMO, and TDO2 can be regarded as the risk prognostic genes for ccRCC.

**Supplementary Information:**

The online version contains supplementary material available at 10.1186/s40001-023-01619-0.

## Introduction

Renal cell carcinoma (RCC) is the most aggressive genitourinary tumor, with an estimated 431,288 new cases of renal cancer and 179,368 death cases in 2020 based on GLOBOCAN 2020 estimates [1]. RCC is a heterogeneous group of tumors, of which ccRCC constitutes approximately 75% of kidney malignancies [1–5]. Despite improvements in cancer screening and diagnostic technologies over the last few decades, approximately 30% of ccRCC patients generally show multiorgan distant metastases at the time of diagnosis and have a poor 5-year outcome; moreover, 20%–40% of patients usually experience a relapse [6–8]. The limited efficacy of chemotherapy and radiotherapy has significantly affected the treatment and management of ccRCC in clinics [9, 10]. In addition, a consensus regimen for the surveillance of ccRCC after nephrectomy is also unavailable, and strict adherence to follow-up guidelines may not be optimally applicable to all patients [11–13]. Abnormalities in the renal metabolism are known to be the principal cause of ccRCC. Therefore, exploration of the pathogenesis of metabolic alterations and identification of the possible prognostic biomarkers driving ccRCC are urgently needed.

The essential amino acid l-tryptophan (Trp), which is exclusively supplemented from dietary sources, serves as a precursor of protein biosynthesis and the generation of crucial bioactive metabolites such as melatonin and serotonin. The kynurenine (Kyn) pathway (KP) is the primary approach for tryptophan metabolism in most mammalian tissues, and is mainly regulated by the rate-limiting enzymes kynurenine monooxygenase (KMO), indoleamine 2,3-dioxygenase (IDO), and tryptophan 2,3-dioxygenase (TDO). Trp and its biologically active metabolites are indispensable for the regulation of a variety of physiological processes, such as immunity, neuronal function, and intestinal homeostasis [14]. Many human disorders, such as cancer, neurodegenerative disease, inflammatory bowel, and cardiovascular disease, are significantly related to imbalances in tryptophan metabolism [15, 16]. A urine metabolomics study showed that tryptophan metabolism represented a critical pathway in ccRCC, while overexpression of tryptophan 2,3-dioxygenase (TDO) in ccRCC was closely related to high levels of Kyn and can predict immunotherapy resistance [17, 18]. Furthermore, specific targeting of tryptophan metabolism-related (TMR) molecules for therapy has attracted growing interest, and some drug candidates targeting TMR genes have even exhibited promise in clinical trials [14, 19]. Although several ongoing clinical studies have explored the metabolic pathways mediated by tryptophan in ccRCC, the clear function of tryptophan metabolism in the progression of ccRCC has not yet been investigated [20].

In this study, genes related to tryptophan metabolism were used to identify stable molecular subtypes, and the distinct characteristics of the clinical, pathway, and immune response were further confirmed. On the basis of these findings, a six-gene-based model for prediction of the prognosis of ccRCC was established, which may provide meaningful guidance for the treatment of ccRCC. Additionally, three risk factors, namely, CYP1B1, KMO, and TDO2, were identified for ccRCC.

## Materials and methods

### Data acquisition and processing

The data for ccRCC copy number variation (CNV) and TCGA-KIRC mutations were downloaded from The Cancer Genome Atlas (TCGA) database (https://portal.gdc.cancer.gov). The ccRCC RNA sequencing (RNA-seq) data were obtained from 481 primary tumor samples and 72 normal kidney samples and were used as training data. Validation data of gene expression profiles of 91 ccRCC samples were obtained from the International Cancer Genome Consortium (ICGC) database (https://icgc.org/).

### Cell culture

Human cancer ACHN cells and normal kidney proximal tubular epithelial HK2 cells were cultured in Dulbecco's modified Eagle’s medium (DMEM; 11,965,084, Gibco) supplemented with 1% penicillin–streptomycin and 10% fetal bovine serum (FBS). The cells were cultivated in humidified incubators with 5% CO_2_ at 37 °C and routinely passaged every 1–2 days.

### Source of genes for tryptophan metabolism

The genes involved in tryptophan metabolism were obtained from the Molecular Signatures Database (MSigDB) (http://www.broadinstitute.org/gsea/msigdb/) 'KEGG_TRYPTOPHAN_METABOLISM'. Forty TMR genes were included, namely, *AADAT, AANAT, ACAT1, ACAT2, ACMSD, AFMID, ALDH1B1, ALDH2, ALDH3A2, ALDH7A1, ALDH9A1, AOC1, AOX1, ASMT, CAT, CYP1A1, CYP1A2, CYP1B1, DDC, ECHS1, EHHADH, GCDH, HAAO, HADH, HADHA, IDO1, IDO2, IL4I1, INMT, KMO, KYNU, MAOA, MAOB, OGDH, OGDHL, TDO2, TPH1, TPH2, WARS1*, and *WARS2*.

### Molecular subtyping related to tryptophan metabolism

To explore the association between TMR genes and ccRCC, a consensus cluster analysis was performed to sort samples into different subtypes according to the expression profiles of the TMR gene [21]. Critical operating parameters included 500 iterations and an 80% resampling rate; resampling of 2 to 10 groups (k = 2 to k = 10) was implemented using the partitioning algorithms around medoids (PAM) and “Spearman” as a distance measure, http://www.bioconductor.org/).

### Construction of a LASSO regression model and survival analysis

The limma R package was applied to identify differentially expressed genes (DEGs) from TMR genes between different subgroups. Univariate Cox regression analysis was developed with the TMR-DEGs and prognostic information to filter out genes most connected to ccRCC prognosis with a false discovery rate (FDR) < 0.05 and |log2 fold change (FC)|> 1. To compress the number of DEGs related to prognosis and construct an optimal prognostic signature, LASSO Cox regression was conducted using the glmnet R package. The Risk Score was calculated using the following formula: Risk Score = Σβ_i_ × Exp_i_, where β indicated the regression coefficient, i represented the variable number, and Exp denoted the levels of gene expression of the variables. A Risk Score of “0” was used as the threshold to classify the samples as high or low risk. Kaplan–Meier survival analysis was performed for measurement of OS.

### Gene set enrichment analysis

We performed gene set enrichment analysis (GSEA) using Hallmark Gene Sets from the Molecular Signatures Database (MSigDB) to identify different functional pathways within the molecular subtypes [22].

### Evaluation of the tumor microenvironment and responses to immunotherapy

The CIBERSORT algorithm (https://cibersort.stanford.edu/) was used to quantify the relative abundance of tumor-infiltrating immune cells in each case of ccRCC. The proportion of immune and stromal cells in the TME was also estimated using the ESTIMATE method (https://sourceforge.net/projects/estimateproject/). The TIDE algorithm (http://tide.dfci.harvard.edu/) was used to assess the response of ccRCC patients to immunotherapy [23].

### Real-time quantitative PCR, tissue microarray, and immunohistochemistry

Immunohistochemistry (IHC) staining was performed in accordance with the standard guidelines to detect the targeted proteins on these 180 tissue specimens. A tissue microarray (TMA) consisting of 150 ccRCC tumor tissues and 30 adjacent normal samples (HKidE180Su02) was purchased from Outdo Biotech (Shanghai, China). The corresponding primary antibodies are shown in Additional file 1: Table S1. The TMA was digitized with a PANNORAMIC 250 scanner (3DHISTECH, Hungary). The outcomes were blindly scored by two pathologists on the basis of the area and intensity of the staining. The samples were separated into high- and low-expression groups in accordance with the median gene expression level. This study was approved by the Ethics Committee of The First Affiliated Hospital, Zhejiang University School of Medicine (No. IIT20220924A). Then, the three identified risky genes were further validated by quantitative real-time polymerase chain reaction (qRT-PCR). Total RNA of the cultured cells was extracted using TRIzol (Invitrogen, CA, USA), and the cDNA was synthesized by the PrimeScript™ RT reagent kit (Takara, Shiga, Japan) in accordance with the manufacturer's instructions. The CFX96 Real-Time PCR Detection Systems (Bio-rad, CA, USA) was employed to perform qRT-PCR to validate the gene expression level, and the specific steps were conducted routinely. All primers used are shown in Additional file 1: Figure S2. Three samples were repeated for each gene to calculate the mean threshold period (Ct), and the relative expression of each mRNA was normalized by the housekeeping gene GAPDH.

### Statistical analysis

All statistical analyses were performed using R version 3.5.1, SPSS v25, and GraphPad Prism 7.0. For assessment of the statistical significance of quantitative data, normally distributed variables were estimated using Student’s t-tests. The Wilcoxon rank-sum test was used for comparisons between groups of non-normally distributed variables. The relationships between two continuous variables were estimated by Spearman's rank order correlation. *P* < 0.05 was defined as significant.

## Results

### Gene mutations and expression changes of TMR genes in ccRCC

**Fig. 1:**
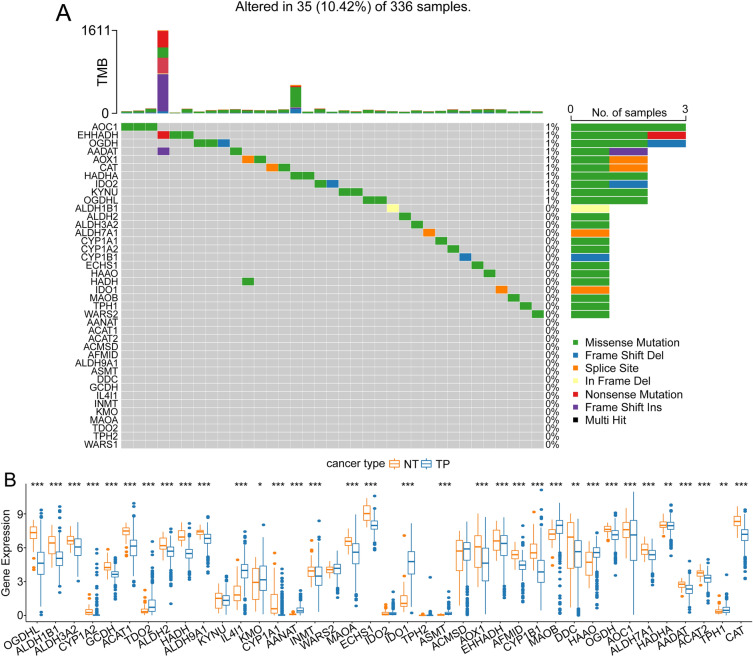
Associations between TMR somatic gene mutations and gene expression in ccRCC. **a** Somatic gene mutation landscape of 40 TMR genes in ccRCC samples. **b** Differences in the expression of 40 TMR genes in tumor tissues from ccRCC patients and adjacent normal tissues

To investigate the genetic alterations in TMR genes that may be associated with the pathogenesis of ccRCC, we evaluated the incidences of genetic alterations in 40 TMR genes among cases from the TCGA ccRCC datasets. Somatic mutations were observed in 35 cases with a detection rate of 10.42% (35/336) (Fig. 1A); the highest mutation frequency was recorded for *AOC1*, followed by *EHHADH*. Since gene alterations are known to influence gene expression, we aimed to clarify the association between the mutations and expression of these 40 TMR genes in ccRCC by evaluating the expression of these 40 TMR genes in ccRCC. In comparison with the normal tissues, the tumor tissues exhibited significantly dysfunctional expression of these TMR genes (Fig. 1B), suggesting that genetic alterations in TMR genes can alter the expression of the corresponding genes and result in a poor prognosis in ccRCC patients.

### Identification of molecular subtypes based on TMR genes

**Fig. 2:**
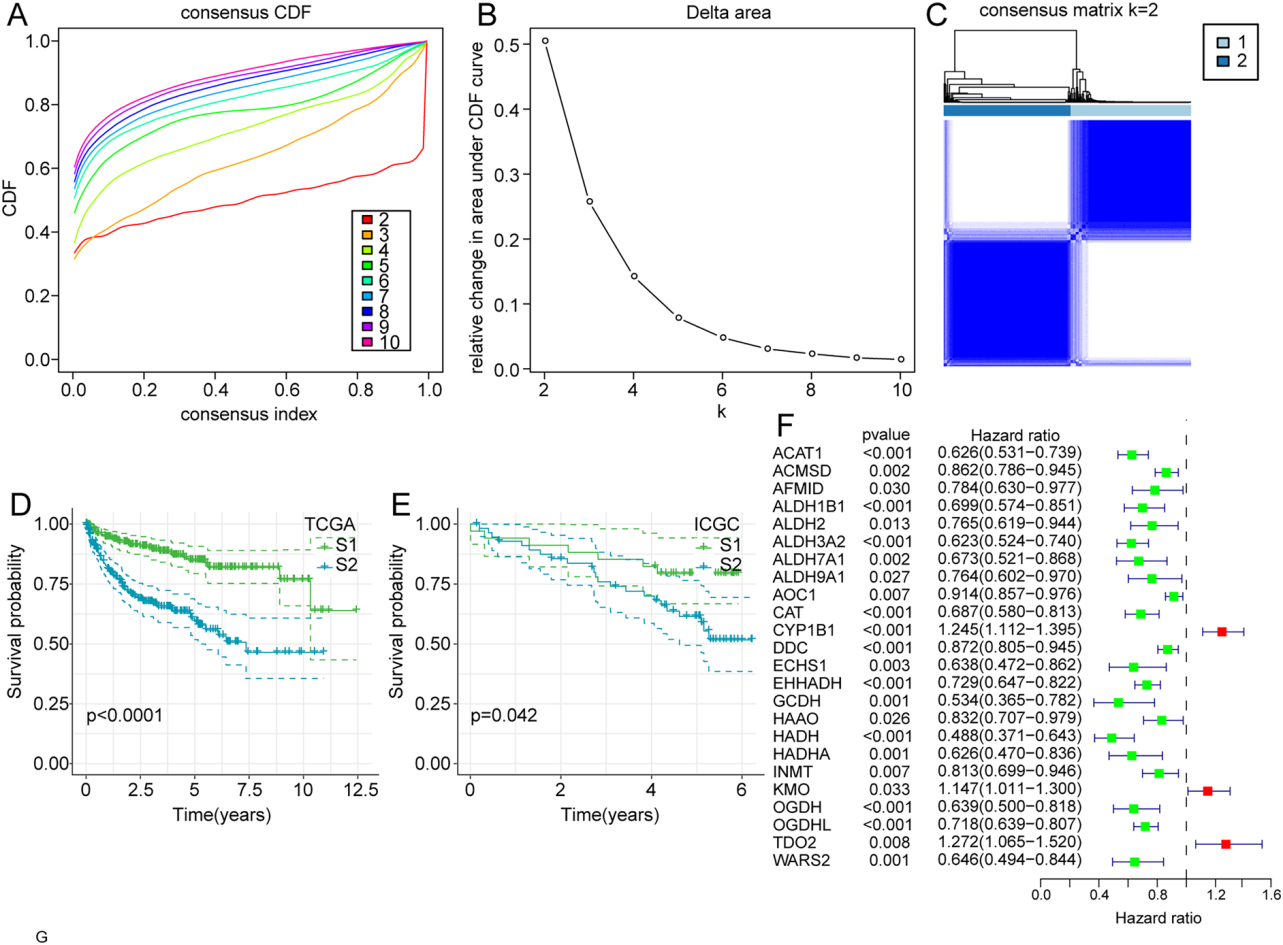
Subgroups of ccRCC divided by TMR genes. **a** CDF curves of the consensus score for k = 2–10 in the TCGA dataset.** b** Relative change in the area under the CDF curve for k = 2–10 in the TCGA dataset. **c** Consensus clustering matrix for k = 2. **d** Kaplan–Meier survival analysis of the patients in the S1 and S2 groups in the TCGA and ICGC datasets. **f** Forest plot of TMR genes in the TCGA dataset

On the basis of the expression levels of the 40 TMR genes, we used consensus clustering to better explore the role of TMR genes in ccRCC and cluster patients into different TMR subgroups. The results confirmed that the stability of the clustering was the most optimal when *k* = 2, when the clustering variable (k) was increased from 2 to 10 (Fig. 2A-C). Thus, ccRCC patients were categorized into two distinct TMR subtypes (named S1 and S2), with 234 patients in cluster 1 and 247 patients in cluster 2. Further, Kaplan–Meier survival analysis revealed that the difference in survival between these two subtypes was significant, and in comparison with the patients in S2, the patients in S1 exhibited significantly better OS (Fig. 2D). The consistent results obtained from the ICGC datasets indicated the high reliability and reproducibility of the molecular subtype based on TMR genes (Fig. 2E). Furthermore, using univariate Cox regression analysis, 24 TMR genes were selected due to their significant correlations with the prognosis of ccRCC. The *CYP1B1*, *KMO*, and *TDO2* genes with a risk ratio greater than 1 were classified as risk factors for ccRCC, whereas the remaining 19/24 genes were considered protective factors (Fig. 2F).

### Upregulation of CYP1B1, KMO, and TDO2 predicts a poor prognosis

**Fig. 3:**
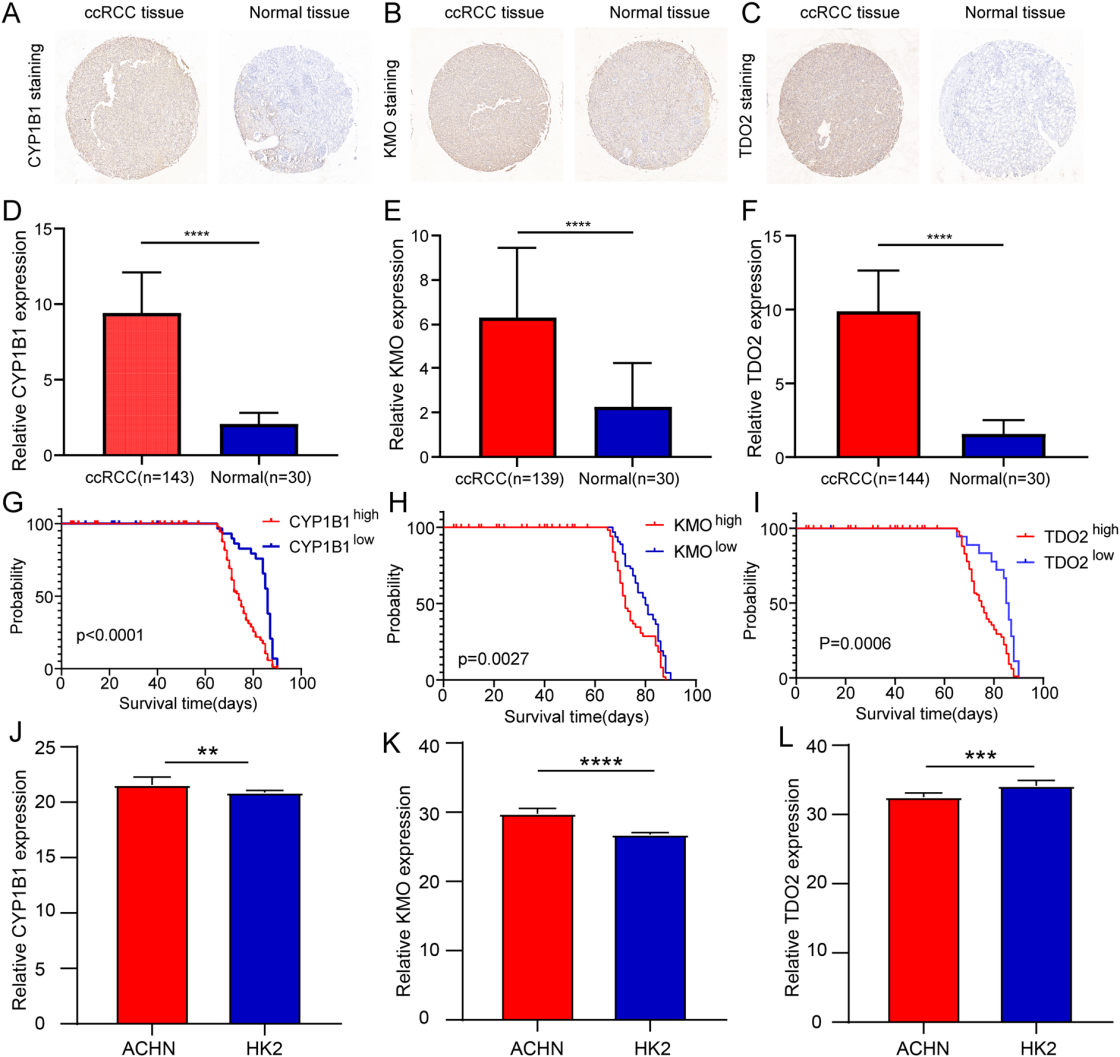
Expression levels of *CYP1B1, KMO*, and *TDO2* and their relationships with the prognosis of ccRCC patients. **a-c** Representative IHC staining of CYP1B1, KMO, and TDO2 in tumor tissues and adjacent normal tissues; scale bar, 200 µm. **d-f** The expression of *CYP1B1, KMO*, and *TDO2* in tumor tissues and adjacent normal tissues. **g-i** Kaplan–Meier survival analysis of the prognosis of patients with ccRCC with low or high expression of *CYP1B1, KMO,* and *TDO2*. **j-l** The expression of CYP1B1, KMO, and TDO2 in the ccRCC cell line (ACHN) and the control cell line (HK2)

To evaluate the relationship of *CYP1B1, KMO*, and *TDO2* with the prognosis of the patients, ICH was performed to explore the levels of CYP1B1, KMO, and TDO2 in tumors and adjacent normal tissues. The results revealed that in comparison with the normal tissues, tumor tissues from ccRCC patients exhibited obviously high CYP1B1, KMO, and TDO2 staining (Fig. 3A-C). The expression of CYP1B1, KMO, and TDO2 in tumor tissues was significantly upregulated in comparison with those in adjacent normal tissues (Fig. 3D-F). The survival analysis further revealed that in comparison with patients with a low density of CYP1B1, KMO, and TDO2, patients with a high density of CYP1B1, KMO, and TDO2 showed significantly poor prognosis (Fig. 3G-I). In addition, we performed qRT-PCR experiments in ccRCC ACHN cell lines and normal HK2 cells for these three genes to validate the microarray results. The results showed that the expression of CYP1B1 and KMO were significantly upregulated in ACHN cells in comparison with those in HK2 cells, whereas TDO2 expression showed the opposite trend (Fig. 3J-L).

### Clinical characteristics, biochemical functions, and immune features of ccRCC molecular subtypes

We explored the distribution of the clinicopathological features of these two ccRCC subtypes. Significant differences in patient sex, T/N/M stage, stage, grade, and survival status were observed between the two subtypes (Additional file 1: Figure S1). We found that S1 consisted of a higher proportion of alive patients with milder disease stages, such as T1, N0, M0, stage I, and early grade. These differences indicated that TMR genes may be involved in ccRCC development by some underlying mechanisms. To assess the pathway enrichment of the two subtypes, we performed GSEA, and the results showed that metastasis-related processes, such as epithelial–mesenchymal transition (EMT), KRAS signaling, and myogenesis, were primarily enriched in S2. Most of the metabolic-related pathways, like fatty acid metabolism, adipogenesis, as well as bile acid metabolism pathways, were enriched in S1 (Additional file 1: Figure: S2A). Further investigation of the oncogenic pathways between the two subtypes showed that S2 had higher scores for WNT, NOTCH, as well as cell cycle-relevant pathways than S1 (Additional file 1: Figure: S2B). To clarify the generality of our subtypes, we explored the distributions of the six immune subtypes proposed by Thorsson et al., of which C3 represented the best prognosis, while C4 correlated with poor outcomes in these two subtypes [24]. As predicted, the results showed that C3 accounted for the predominant proportion in S1 compared to S2, which was in agreement with our results showing that S1 had a better survival (Additional file 1: Figure S3A). We also conducted an analysis of the immune landscape between the 2 molecular subtypes. Additional file 1: Figure S3B shows the top 10 gene mutations with significant differences between the 2 molecular subtypes, including VHL, PBRM1, TTN, SETD2, MTOR, MUC16, KDM5C, DNAH9, and ATM.

**Fig. 4:**
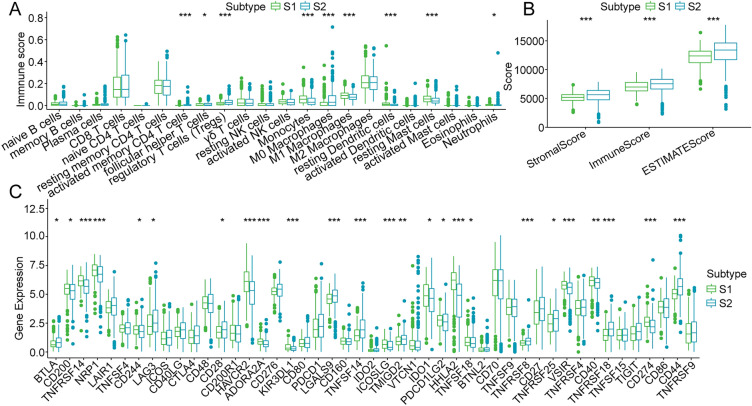
Differences in immune characteristics between patients in the S1 and S2 groups. Differences in the distributions of immune cell infiltration (**a)**, immune scores (**b**), and expression of immune checkpoint genes **(c)** between patients of the S1 and S2 groups

Since the significantly different gene functions between the two TMR subtypes mainly focused on immune- or metabolism-related pathways, we speculated that this phenomenon might be related to the different immune microenvironments between the two TMR subtypes. Therefore, we assessed the immune infiltration analysis data to further explore the influence of TMR genes on the tumor immune microenvironment of ccRCC. TME is known to play a crucial role in tumorigenesis and treatment, so we further investigated the differences in immunological characteristics between the two subtypes. A CIBERSORT analysis was performed to explore the levels of the 22 types of immune cells that infiltrated the tumors in the two subtypes. The results showed a clear difference in the proportions of immune cells between the two subtypes. Specifically, the S1 group showed fewer follicular helper T cells, activated CD4 memory T cells, regulatory T cells, and M0 macrophages and more monocytes, neutrophils, M1 macrophages, resting dendritic cells, and resting mast cells (Fig. 4A). The distinctly lower immune, stromal, and estimate scores in S1 suggested that S1 possessed relatively higher tumor purity (Fig. 4B). Considering the distinct TMEs of the two subtypes, we examined the responses of the patients in the two subtypes to immunotherapies. The expression of the immune checkpoint is gradually considered to reflect the patients’ response to immunotherapy in cancer, and most of the immune checkpoint-associated genes were observed to be differentially expressed in the two subtypes. In comparison with their expression levels in S2, most of the immune checkpoints, such as *CD200, TNFRSF14, NRP1, CD244, HAVCR2, ADORA2A, KIR3DL1, ICOSLG, IDO1, HHLA2, TNFSF18, VSIR, CD40,* and *CD274* were significantly overexpressed in S1, while the expressions of *BTLA, LAG3, CD28, LGALS9, TNFSF14, TMIGD2, PDCD1LG2, TNFRSF8, TNFRSF25, TNFRSF18,* and *CD44* were decreased in S1 (Fig. 4C).

### Construction of a prognostic Risk Score relevant to tryptophan metabolism for ccRCC patients

**Fig. 5:**
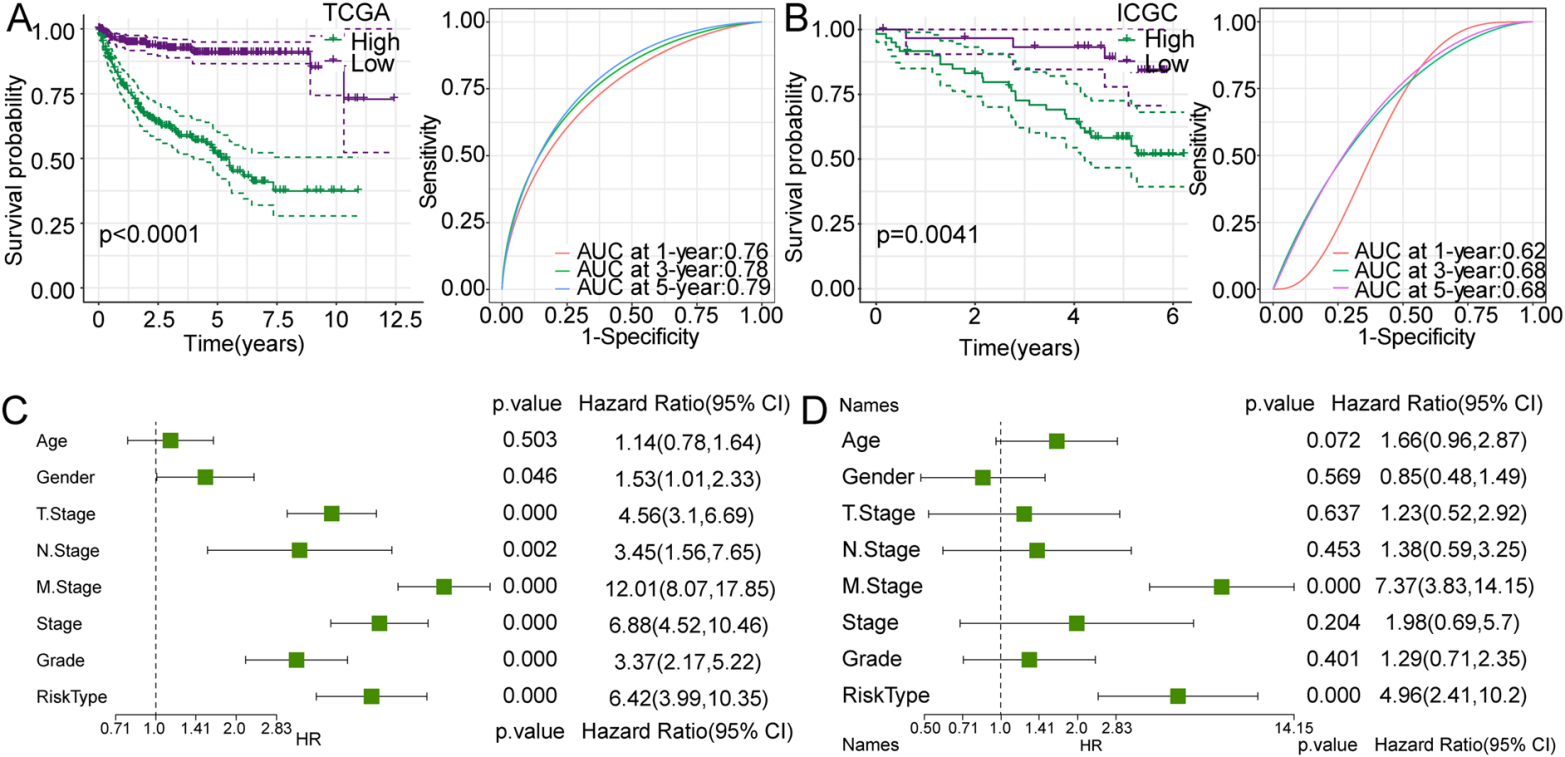
Identification and validation of the predictive abilities of the six-gene model in low- and high-risk groups. **a-b** Kaplan–Meier survival and receiver operating characteristic (ROC) curves of the six-gene model in the TCGA and ICGC datasets. **c-d** Univariate and multivariate Cox regression analysis for the prognosis of patients with ccRCC

In previous analyses, we demonstrated the presence of differences in OS, TME, and immunotherapy sensitivity between S1 and S2. Next, we chose the DEGs from two subtypes to build a prognostic model for ccRCC patients. In DEG analysis of S1 and S2, 426 DEGs were screened, including 372 upregulated genes and 54 downregulated genes (Additional file 1: Figure S4A). The LASSO regression analysis further compressed the gene number recruited into the prognostic model. After combining the variation trajectories of each independent variable and performing tenfold cross-validation for the model, 0.037 was selected as the optimal lambda value and 14 genes were obtained (Additional file 1: Figure S4B). We then minimized the Akaike information criteria (AIC) to generate the most stable model consisting of six genes (*ENAM, DHDH, SHISA9, CTHRC1, CRABP2*, and *IL20RB*) (Additional file 1: Figure S4C). The Risk Score was established by combining each gene expression value multiplied by the respective coefficient as follows: Risk Score = −0.366 × ENAM-0.195 × DHDH-0.231 × SHISA9 + 0.095 × CTHRC1 + 0.083 × CRABP2 + 0.068 × IL20RB. The median value was employed to classify cases into high- and low-risk groups. Furthermore, we compared the association between risk groups and molecular subtypes and found that the majority of S2 patients with a poorer prognosis belonged to the high-risk group (Additional file 1: Figure S4D). As shown in Fig. 5A, in comparison with patients of the low-risk group, patients of the high-risk group exhibited poor OS rates, and the areas under the curve (AUCs) for the 1-, 3- and 5-year OS in were 0.76, 0.78, and 0.79, respectively (Fig. 5A), indicating the favorable predictive capacity of the prognostic model for patient survival rates. Furthermore, to reduce bias, a verification analysis for this six-gene-based model was performed using the ICGC cohort. Similar results showed that the OS rates of the high-risk group were inferior to those of the low-risk group and the AUCs for the OS of 1-, 3-, and 5- year were 0.62, 0.68, and 0.68 (Fig. 5B). Taken together, these results indicated that the established six-gene model displayed robust survival prediction efficacy for ccRCC patients. The univariate and multivariate Cox regression analysis further verified the independence for the prognosis of Risk Score, demonstrating the potential predictive performance of the six-gene model in clinical practice (Fig. 5C-D).

### The correlation of Risk Score with clinical characteristics and pathway signatures

To explore the clinical applicability of Risk Score, we also explored the relationships of Risk Score with clinical characteristics in the TCGA dataset. The results showed a remarkably different distribution of clinical characteristics such as sex, TNM stage, stage, grade, and survival status, except for age, between the high- and low-risk groups (Additional file 1: Figure S5). Patients with a worse clinical stage showed a higher Risk Score. Collectively, these results showed a strong correlation between clinical features and the six-gene-based model, suggesting that a higher Risk Score usually represented a high degree of malignancy. Next, we applied ssGSEA to compute the enrichment of the pathways in each sample and obtained a total of seven key pathways with correlation values greater than 0.4. Five of these metabolism-related pathways were inversely associated with the Risk Score, while two showed positive relationships with the Risk Score (Additional file 1: Figure S5).

### Relationship between Risk Score and tumor immune characteristics

**Fig. 6:**
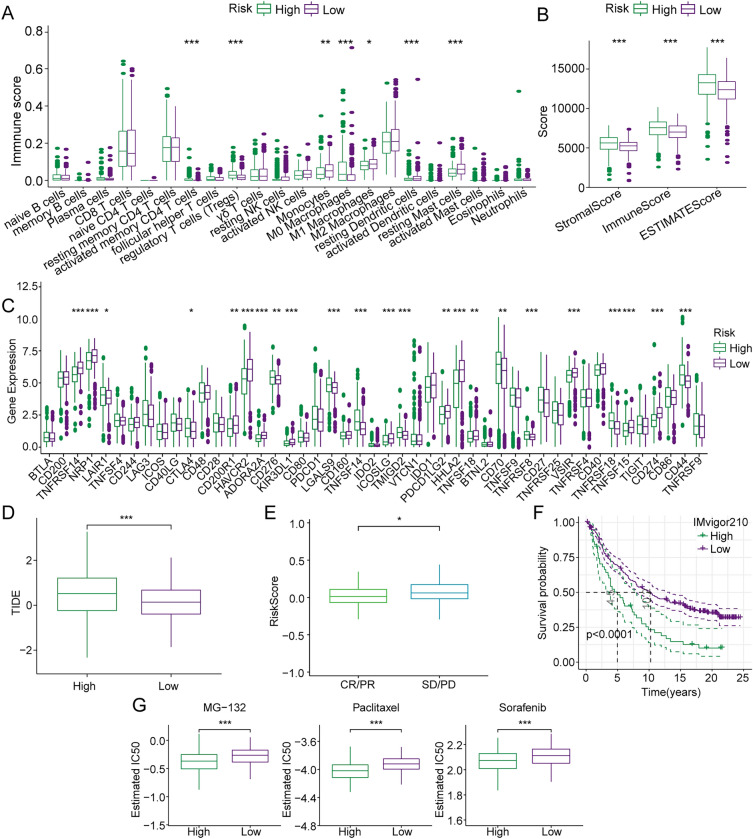
Relationships of Risk Score with tumor immune characteristics and clinical application. Differences in the distributions of immune cells (**a)**, immune scores **(b)**, expression of immune checkpoint genes **(c)**, TIDE score **(d),** and therapeutic response state **(e)** between low- and high-risk groups. **f** Kaplan–Meier survival analysis of patients in the low- and high-risk groups in the IMvigor210 dataset. **g** Estimation of drug sensitivity between the low- and high-risk groups

To elucidate the association of the Risk Score with TME, the CIBERSORT algorithm was used to quantify the relative levels of 22 immune cell types in the high- and low-risk groups. The proportions of the infiltrated immune cells were remarkably distinct in the two groups, with the high-risk group showing notably higher levels of Tregs and M0 macrophages and fewer M1 macrophages (Fig. 6A). The higher ESTIMATE scores in the high-risk group reflected the relatively lower tumor purity in the high-risk group of patients (Fig. 6B). Thus, the Risk Score was strongly associated with the ccRCC TME. Considering the close relationship between the Risk Score and immune activity, we next compared the levels of immune checkpoint genes in the two groups and found that patients in the high-risk group had lower levels of *TNFRSF14, NRP1, CD200R1, HAVCR2, ADORA2A, KIR3DL1, ICOSLG, HHLA2, TNFSF15,* and *CD274* (Fig. 6C). Subsequently, the TIDE algorithm was used to illustrate the predictive performance of Risk Score for immunotherapy. The TIDE scores were higher in the high-risk group; thus, patients in this group showed a greater tendency toward escape from antitumor immune activity and unfavorable responses to immunotherapy (Fig. 6D). We also assessed the prognostic efficacy of Risk Score in the IMvigor210 cohort, and patients who received anti-PD-L1 treatment showed stable disease (SD)/progressive disease (PD) with a significantly higher Risk Score than those with complete response (CR)/partial response (PR) (Fig. 6E). The survival analysis also showed that patients in the high-risk group had a poorer OS than those in the low-risk group (Fig. 6F). Furthermore, since chemotherapy is also a traditional therapeutic approach for ccRCC, we estimated the IC50 of the commonly used chemotherapeutic agents for ccRCC (MG-132, paclitaxel, and sorafenib) in the two groups. We observed that patients in the high-risk group showed lower IC50 values, indicating that these three chemotherapeutic agents were more beneficial to patients in the high-risk group (Fig. 6G). These results confirmed that Risk Score could identify the susceptibility of patients to various types of immunotherapies and chemotherapies.

## Discussion

Metabolic reprogramming, especially tryptophan metabolism, has been increasingly shown to have significant associations with the progression of various cancers [25–28]. RCC is fundamentally a metabolic disease characterized by reprogramming of energy metabolism, involving multiple metabolic pathways including glycolysis, mitochondrial bioenergetics, lipid metabolism, and amino acid metabolism [29–32]. Particularly, RCC exhibits a rerouting of metabolic alterations through glycolysis, favoring energy production via this pathway instead of oxidative phosphorylation to efficiently utilize glucose to meet their growth and proliferation demands [33–35]. Additionally, mitochondrial bioenergetics and oxidative phosphorylation capacity are compromised in RCC cells. Mitochondria, the primary organelles responsible for energy production, exhibit abnormal function in RCC, potentially leading to unstable energy supply [33, 36–38]. Furthermore, lipid metabolism is dysregulated in RCC. Abnormal lipid uptake and metabolism contribute to lipid accumulation and aberrant signaling pathways in RCC cells [33, 36–38]. Amino acid metabolism, particularly tryptophan and its metabolite kynurenine, is also altered in RCC. Tryptophan metabolism was reported to contribute to the tumorigenesis of various malignancies, mainly by inhibiting the antitumor immune response and improving the malignant properties of cancer cells [39, 40]. Altered tryptophan metabolism has been shown to have important implications for ccRCC [41]. Wettersten HI et al. indicated that ccRCC patients exhibited significantly elevated levels of immunosuppressive tryptophan metabolites, especially quinolinate and Kyn, and the upregulation of tryptophan metabolism is closely correlated with the grade of the ccRCC tumor [42, 43]. Lucarelli, G. et al*.* demonstrated for the first time that activation of KP portended adverse cancer-specific survival (CSS) and progression-free survival (PFS) in ccRCC and that the Kyn-to-tryptophan ratio could be used to reflect the aggressiveness of ccRCC [44].

Furthermore, IDO, the rate-limiting enzyme of the Kyn pathway, was shown to perform crucial functions in the induction of immune tolerance and is associated with the long-term survival of patients with ccRCC [45]. Selective inhibition of IDO has been increasingly shown to prevent its immunosuppressive effect on antitumor T cell activation, which is currently being tested in preclinical models of ccRCC [46, 47]. RCC is characterized by a high degree of immune infiltration, and the immune microenvironment plays a crucial role in regulating angiogenesis and abnormal inflammatory features [48–50]. Recent evidence suggests that the activation of specific metabolic pathways is closely associated with these processes [44, 51]. And the immune microenvironment features within RCC TME significantly impact the biological characteristics of RCC cells and affect systemic treatment efficacy [52–56]. Therefore, understanding and intervening in the immune microenvironment features within RCC TME are of great significance for the treatment of RCC. In our study, we employed immune infiltration analysis to investigate the impact of TMR gene on the tumor immune microenvironment in ccRCC. The results revealed distinct immunological characteristics and significant differences in the proportions of 22 immune cell types between the two subtypes of tumors. Furthermore, differential expression of most immune checkpoint-related genes was observed between the two subtypes. Additionally, our study demonstrated a close association between risk scores and immune microenvironment characteristics in ccRCC. Importantly, in the risk score model based on TMR genes, high-risk group patients exhibited a greater tendency towards tumor immune evasion. These findings confirm the efficacy of risk scoring based on TMR genes in predicting the response of RCC patients to immunotherapy.

Polygenic risk models have become widespread in several cancers and have begun to make an impact in individualized disease risk predictions and clinical management of patients [57–59]. Wang Z et al. identified an immune-related prognostic model for HCC and its close relationship with the status of the tumor immune microenvironment [60]. They also developed a prognostic classifier based on genes related to lipid metabolism for clinical application in breast invasive carcinoma [61]. Lin X et al. presented novel multigene panels that could be used to assess the relapse and fatality risk stratification of adrenocortical carcinoma [62]. Cui Y et al. merged the two prognosis models based on infiltrated tumor immune cells and ceRNA networks in ccRCC to improve the personalized management of ccRCC patients [63]. Dong Y et al*.* validated the association of ferroptosis with tumor immunity in ccRCC and constructed a risk model based on ferroptosis-associated lncRNAs for predicting the prognosis in patients with ccRCC [64]. However, these models did not concomitantly clarify the overall immune status of patients and practically translated into clinical applications. In our study, we identified two molecular subtypes of ccRCC on the basis of the expression profiles of 40 TMR genes, which exhibited distinct differences in patient prognoses, clinical characteristics, pathway characteristics, and even immune characteristics. These studies collectively support the crucial role of tryptophan metabolism in the prognosis of ccRCC and its significant influence on immunotherapies.

Meanwhile, our study had several limitations. Since we did not conduct in-depth research of the mechanism, the underlying molecular mechanisms of carcinogenesis induced by CYP1B1, KMO, and TDO2 remain to be clarified. Additional studies are warranted to elucidate these mechanisms, which may contribute greatly to ccRCC development. Additionally, our findings depended largely on public databases using bioinformatics analysis; therefore, further large-sample studies are required to verify these findings.

In conclusion, this study indicate that our six-gene-based risk model based on tryptophan metabolism can be used to predict ccRCC prognosis and determine the clinical response of patients to treatment, especially immunologic drugs and subsequent individualized treatments. Furthermore, high expression levels of *CYP1B1, TDO2*, and *KMO* were correlated to a poor outcome in ccRCC patients.

### Supplementary Information

**Additional file 1: Figure S1.** Relationships of subgroups with clinical characteristics. **Figure S2.** Relationships of subgroups with functional pathways (**a**) and oncogenic pathways (**b**). **Figure S3.** Relationships of subgroups with pan-cancer immune molecular subtypes (**a**) and immune landscapes (**b**). **Figure S4.** Construction of a TMR prognosis model. **a** Volcano plot of DEGs between patients of the S1 and S2 groups. **b** Cross-validation for selection of parameters to adjust in the LASSO model. **c** Penalty plot for the LASSO model for the six genes. **d** The association between risk groups and molecular subtypes. **Figure S5.** Relationships of Risk Score with clinical characteristics. **Figure S6.** Relationships of Risk Score with clinical characteristics and functional pathways.

**Additional file 2: Table S1.** Primers of 3 genes for RT-qPCR.

**Additional file 3: Table S2.** Primary antibody information of 3 genes for IHC.

## Data Availability

The data that support the findings of this study are openly available from The Cancer Genome Atlas database (https://portal.gdc.cancer.gov/).
