# Supplementary material for: Comprehensive analysis of a tryptophan metabolism-related model in the prognostic prediction and immune status for clear cell renal carcinoma
Source: Eur J Med Res. 2024 Jan 5;29:22. doi: 10.1186/s40001-023-01619-0 (PMC10768089; doi:10.1186/s40001-023-01619-0)
Supplement: Supplementary file 1 — Additional file 1: Figure S1. Relationships of subgroups with clinical characteristics. Figure S2. Relationships of subgroups with functional pathways (a) and oncogenic pathways (b). Figure S3. Relationships of subgroups with pan-cancer immune molecular subtypes (a) and immune landscapes (b). Figure S4. Construction of a TMR prognosis model. a Volcano plot of DEGs between patients of the S1 and S2 groups. b Cross-validation for selection of parameters to adjust in the LASSO model. c Penalty plot for the LASSO model for the six genes. d The association between risk groups and molecular subtypes. Figure S5. Relationships of Risk Score with clinical characteristics. Figure S6. Relationships of Risk Score with clinical characteristics and functional pathways. [file 40001_2023_1619_MOESM1_ESM.docx]

**Figure S1.**

**
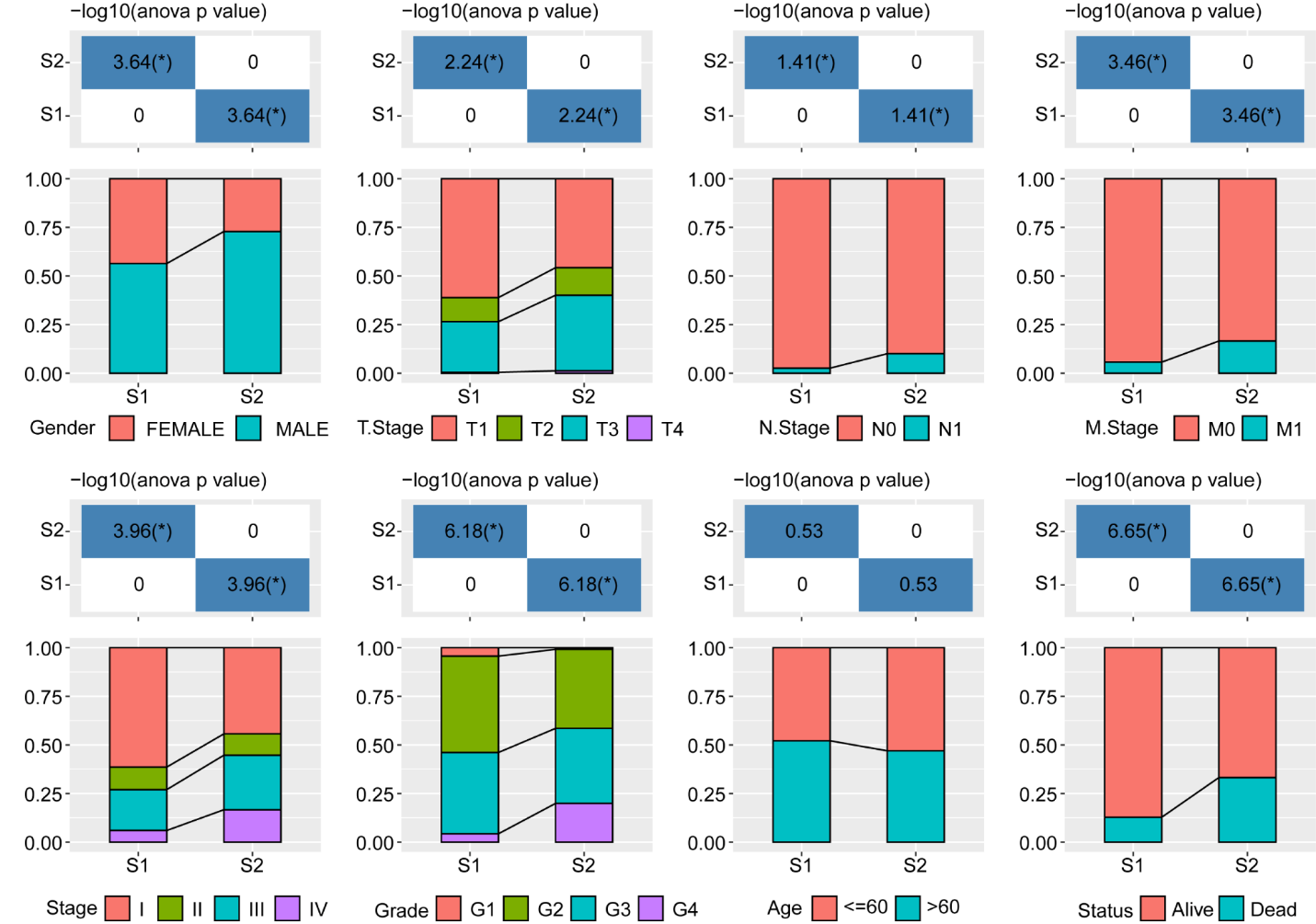
**

Relationships of subgroups with clinical characteristics.

**Figure S2.**

**
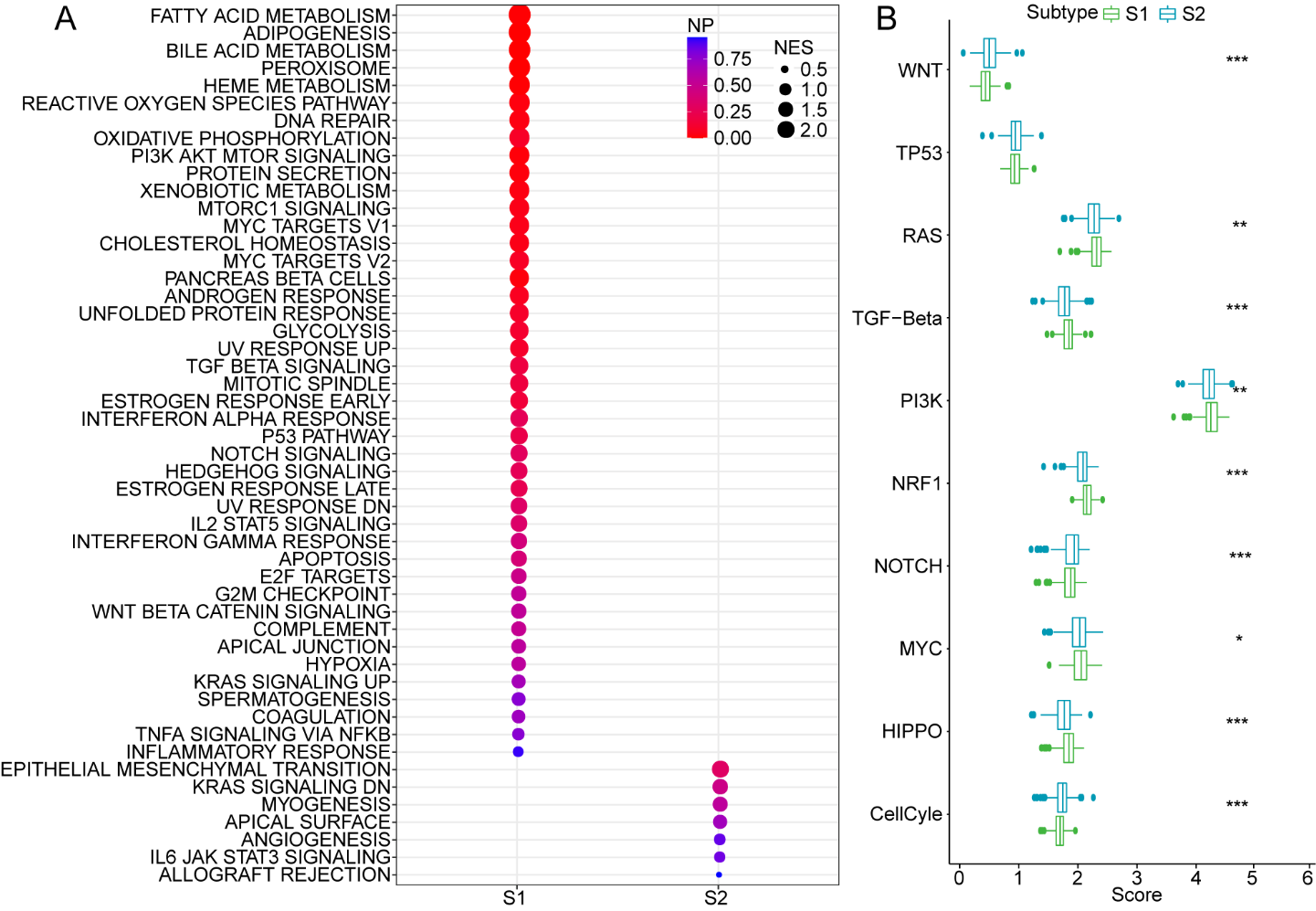
**

Relationships of subgroups with functional pathways (a) and oncogenic pathways (b).

**Figure S3.**

**
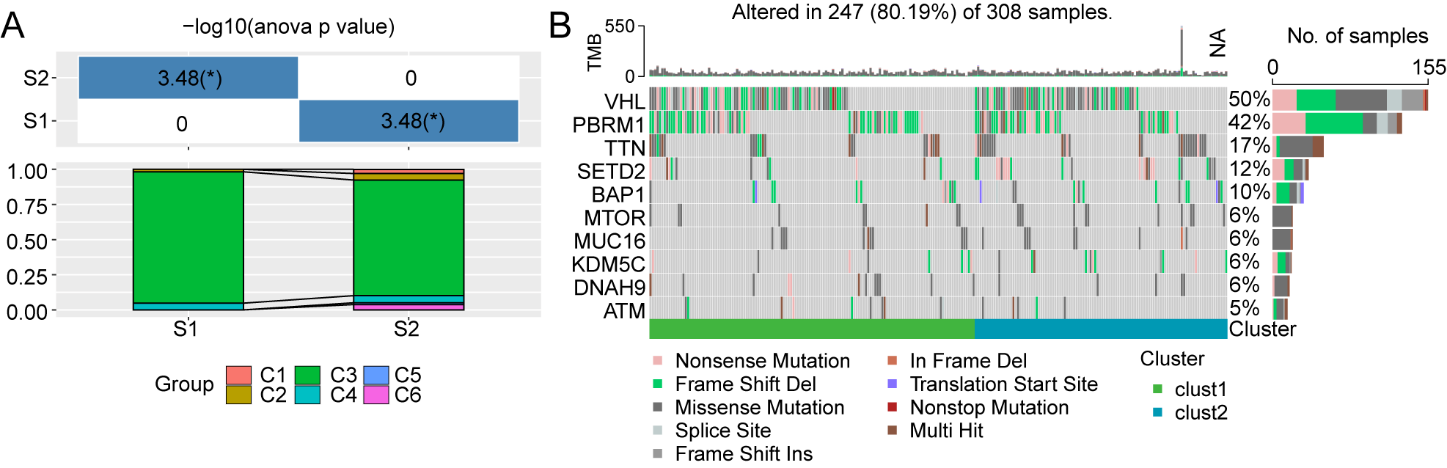
**

Relationships of subgroups with pan-cancer immune molecular subtypes (a) and immune landscapes (b).

**Figure S4.**

**
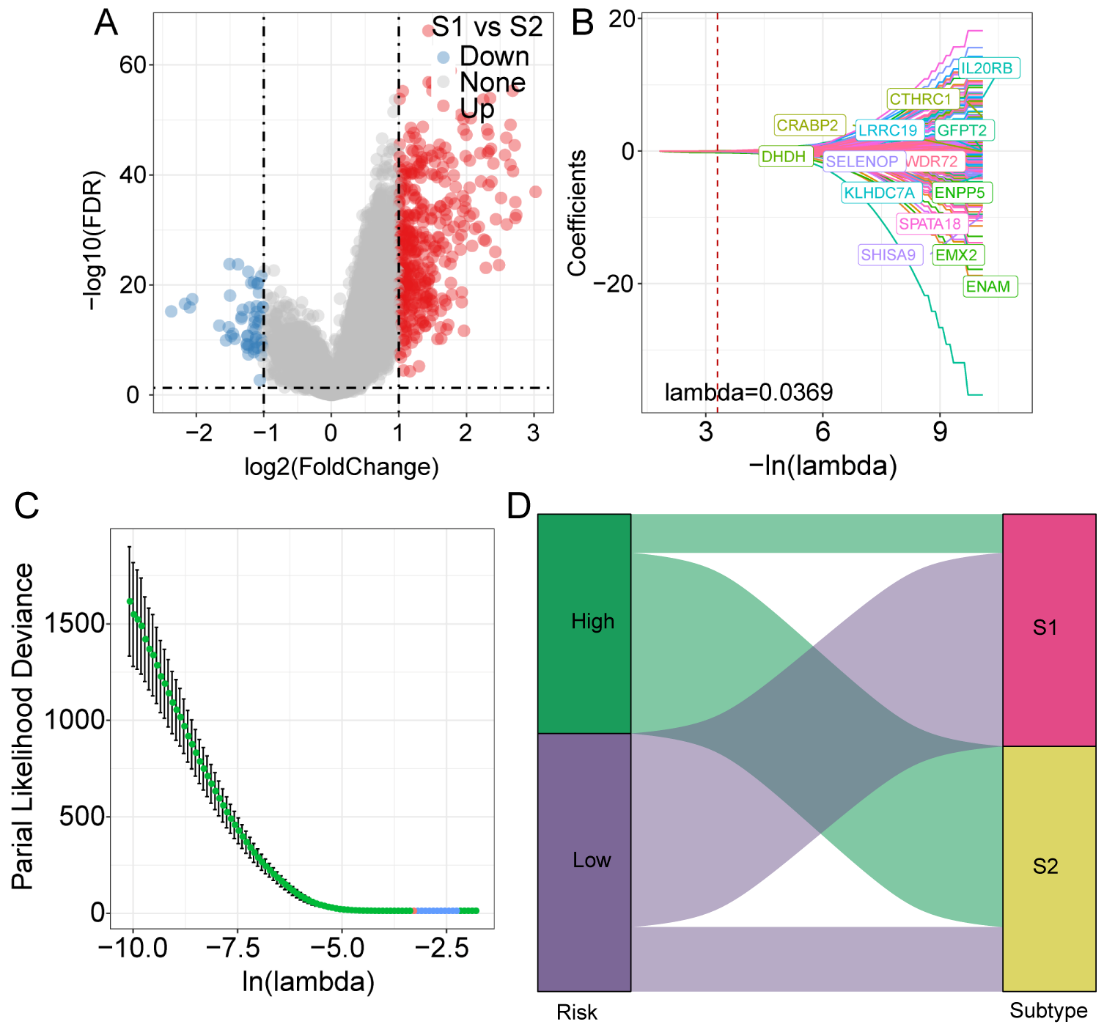
**

**Construction of a TMR prognosis model. (a)** Volcano plot of DEGs between patients of the S1 and S2 groups. **(b)** Cross-validation for selection of parameters to adjust in the LASSO model. **(c)** Penalty plot for the LASSO model for the six genes. **(d)** The association between risk groups and molecular subtypes.

**Figure S5.**

**
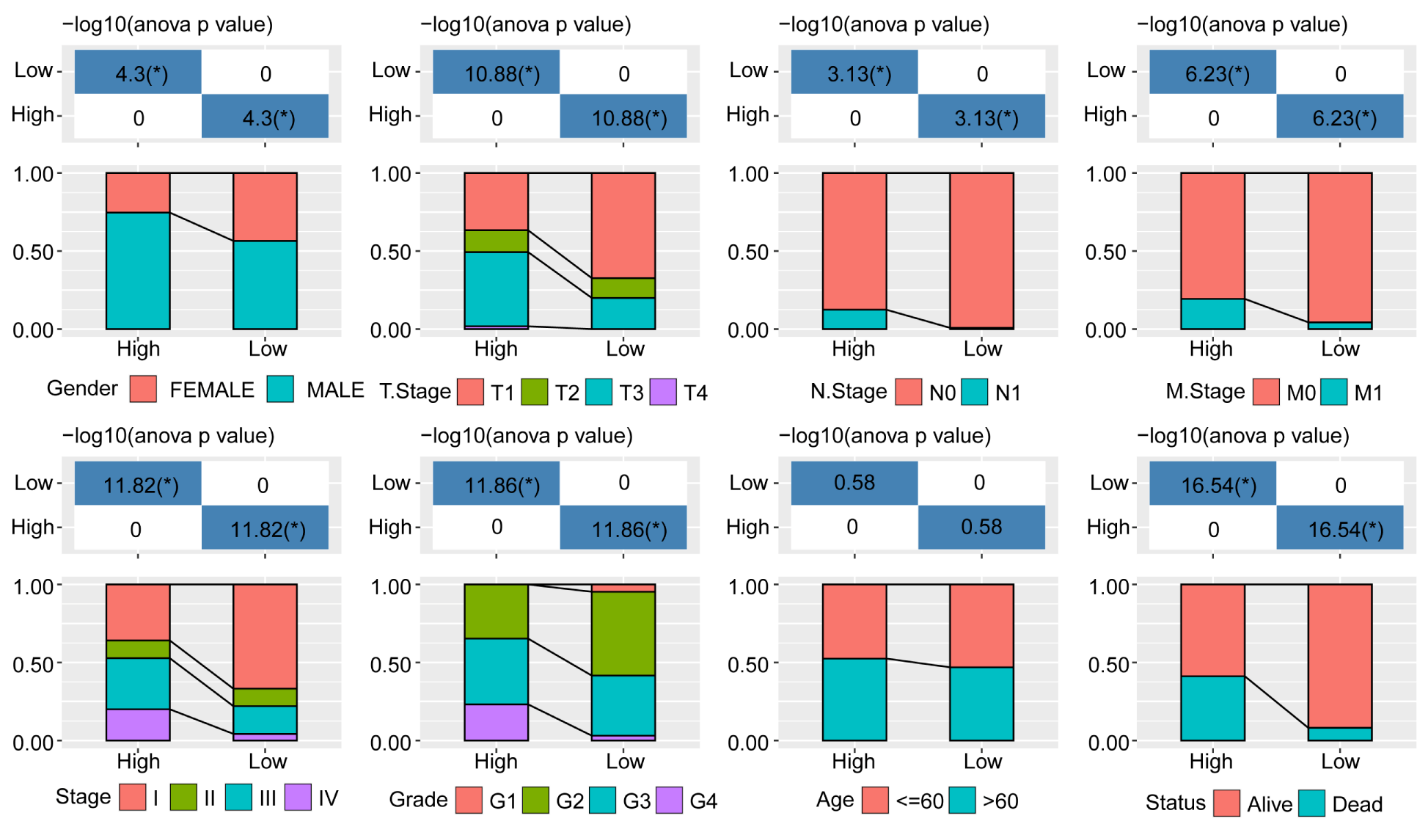
**

Relationships of Risk Score with clinical characteristics.

**Figure S6.**

**
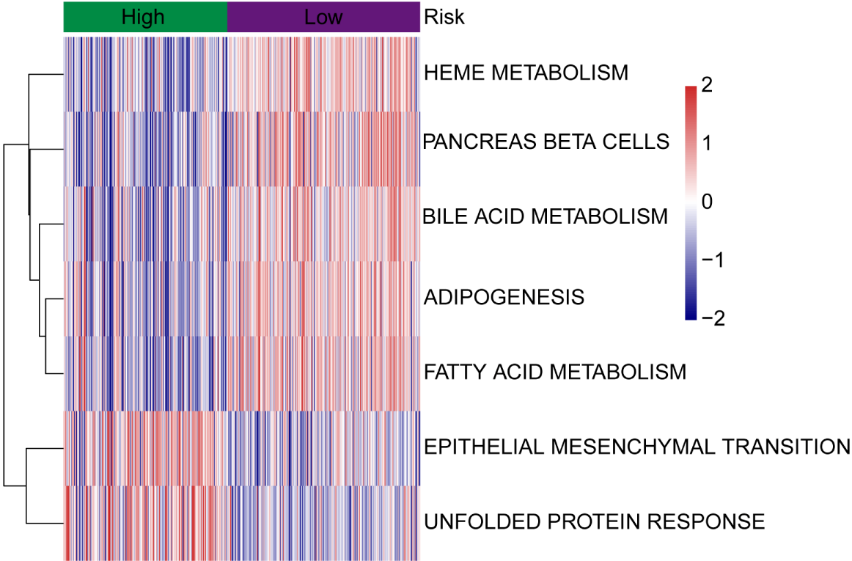
**

Relationships of Risk Score with clinical characteristics and functional pathways.
