# Supplementary material for: Comprehensive analysis of a tryptophan metabolism-related model in the prognostic prediction and immune status for clear cell renal carcinoma
Source: Eur J Med Res. 2024 Jan 5;29:22. doi: 10.1186/s40001-023-01619-0 (PMC10768089; doi:10.1186/s40001-023-01619-0)
Supplement: Supplementary file 2 — Additional file 2: Table S1. Primers of 3 genes for RT-qPCR. [file 40001_2023_1619_MOESM2_ESM.docx]

| Table S1. Primers of 3 genes for RT-qPCR. | |
| --- | --- |
| Target | Sequence (5'–3') |
| CYP1B1(F) | AAGTTCTTGAGGCACTGCGAA |
| CYP1B1(R) | GGCCGGTACGTTCTCCAAAT |
| KMO(F) | TAGCCCTTTCTCATAGAGGACG |
| KMO(R) | CTCTCATGGGAATACCTTGGGA |
| TDO2(F) | AAGGTTGTTTCTCGGATGCAC |
| TDO2(R) | TGTCATCGTCTCCAGAATGGAA |
