# Supplementary material for: Comprehensive analysis of a tryptophan metabolism-related model in the prognostic prediction and immune status for clear cell renal carcinoma
Source: Eur J Med Res. 2024 Jan 5;29:22. doi: 10.1186/s40001-023-01619-0 (PMC10768089; doi:10.1186/s40001-023-01619-0)
Supplement: Supplementary file 3 — Additional file 3: Table S2. Primary antibody information of 3 genes for IHC. [file 40001_2023_1619_MOESM3_ESM.docx]

| Table S2. Primary antibody information of 3 genes for IHC. | | | |
| --- | --- | --- | --- |
| Primary antibody | Supplier | Product code | Dilution factor |
| Anti-CYP1B1 | Abcam | ab185954 |  |
| Anti-KMO | Proteintech | 10698-1-AP |  |
| Anti-TDO2 | Proteintech | 15880-1-AP |  |
